# Accurate identification of Culicidae at aquatic developmental stages by MALDI-TOF MS profiling

**DOI:** 10.1186/s13071-014-0544-0

**Published:** 2014-12-02

**Authors:** Constentin Dieme, Amina Yssouf, Anubis Vega-Rúa, Jean-Michel Berenger, Anna-Bella Failloux, Didier Raoult, Philippe Parola, Lionel Almeras

**Affiliations:** Aix Marseille Université, Unité de Recherche en Maladies Infectieuses et Tropicales Emergentes (URMITE), UM63, CNRS 7278, IRD 198 (Dakar, Sénégal), Inserm 1095, WHO collaborative center for rickettsioses and other arthropod borne bacterial diseases, Faculté de Médecine, 27 bd Jean Moulin, 13385 Marseille cedex 5, France; Laboratoire d’Ecologie Vectorielle et Parasitaire, Université Cheikh Anta Diop de Dakar, Dakar, Senegal; Department of Virology, Institut Pasteur, Arboviruses and Insect Vectors, Paris, France

**Keywords:** MALDI-TOF mass spectrometry, Culicidae, Aquatic stages, Species identification, Vectors

## Abstract

**Background:**

The identification of mosquito vectors is generally based on morphological criteria, but for aquatic stages, morphological characteristics may be missing, leading to incomplete or incorrect identification. The high cost of molecular biology techniques requires the development of an alternative strategy. In the last decade, matrix-assisted laser desorption/ionization time-of-flight mass spectrometry (MALDI-TOF MS) profiling has proved to be efficient for arthropod identification at the species level.

**Methods:**

To investigate the usefulness of MALDI-TOF MS for the identification of mosquitoes at aquatic stages, optimizations of sample preparation, diet, body parts and storage conditions were tested. Protein extracts of whole specimens from second larval stage to pupae were selected for the creation of a reference spectra database. The database included a total of 95 laboratory-reared specimens of 6 mosquito species, including *Anopheles gambiae* (S form), *Anopheles coluzzi* (M form), *Culex pipiens pipiens*, *Culex pipiens molestus*, *Aedes aegypti* and 2 colonies of *Aedes albopictus*.

**Results:**

The present study revealed that whole specimens at aquatic stages produced reproducible and singular spectra according to the mosquito species. Moreover, MS protein profiles appeared weakly affected by the diet provided. Despite the low diversity of some MS profiles, notably for cryptic species, clustering analyses correctly classified all specimens tested at the species level followed by the clustering of early *vs*. late aquatic developmental stages. Discriminant mass peaks were recorded for the 6 mosquito species analyzed at larval stage 3 and the pupal stage. Querying against the reference spectra database of 149 new specimens at different aquatic stages from the 6 mosquito species revealed that 147 specimens were correctly identified at the species level and that early and late developmental stages were also distinguished.

**Conclusions:**

The present work highlights that MALDI-TOF MS profiling may be useful for the rapid and reliable identification of mosquito species at aquatic stages. With this proteomic tool, it becomes now conceivable to survey mosquito breeding sites prior to the mosquitoes’ emergence and to adapt anti-vectorial measures according to the mosquito fauna detected.

**Electronic supplementary material:**

The online version of this article (doi:10.1186/s13071-014-0544-0) contains supplementary material, which is available to authorized users.

## Background

Mosquito-borne diseases (MBDs) are a major public health problem leading to millions of human deaths each year [1]. Although vaccines, therapeutics or prophylaxis exist for some MBDs, the best method to protect against MBDs is to avoid mosquito bites [2]. To this end, several methods have been developed to prevent mosquito bites with personal protective measures and the implementation of vector control strategies [3]. Long-lasting impregnated nets (LLINs) and indoor-residual spraying (IRS) directed against the adult vector population, for example, have been shown to efficiently decrease MBDs transmitted by nocturnal and indoor mosquitoes such as *Anopheles-*malaria vectors [4]. Nevertheless, these control strategies are less efficient against mosquitoes with outdoor and/or diurnal biting activities such as the *Aedes* genus, vectors of dengue fever, yellow fever, and chikungunya, or the *Culex* genus, vectors of West Nile virus, Japanese encephalitis and St. Louis encephalitis [5]. An alternative strategy is to decrease adult mosquito densities by targeting their aquatic stages, which can be achieved by reducing vector larval habitats or with the application of chemical or biological agents to kill the larvae [3]. These anti-vectorial measures target both outdoor and indoor mosquito vectors with distinct circadian biting activities. Moreover, in contrast with adult mosquitoes, which are highly mobile flying insects and can escape many intervention measures, mosquito larval and pupal developmental stages are confined to aquatic habitats (*i.e.,* breeding sites) and cannot readily evade control interventions [6]. Because a single area could harbor several mosquito species differing in their vector competences, host-feeding preferences or larval habitat requirements [7], a correct taxonomic classification is crucial to distinguish vectors from non-vectors. In addition, to evaluate the impact of control measures, a precise determination of the abundance and proportion of the various mosquito species is needed both before and after implementation of the vector control program.

Classically, mosquitoes are identified using morphological criteria. Nevertheless, this method is time-consuming, requiring entomological expertise and training, and may lead to misidentifications, particularly for closely related species (*e.g.,* species complex) [8]. This problem is exacerbated in aquatic stages (*i.e.,* larval or pupal stages), where identification keys or morphological characteristics may be missing, leading to incomplete or incorrect identification. Alternatively, larvae can be reared until the imago stage for which morphological characters are better established. Nevertheless, this time-consuming strategy is incompatible with field constraints requiring the rapid and accurate identification of mosquitos to monitor and adapt anti-vectorial measures.

To circumvent these problems, polymerase chain reaction (PCR) and other molecular biological methods have been increasingly applied for the identification of mosquitoes [9,10]. Despite the development of a universal standard method serving as a “barcode” for the identification of organisms including insects [11,12], Foster and collaborators have reported the limitations of using a single gene for mosquito identification [13]. These authors underlined the need to combine several genes for the accurate identification of mosquitoes such as the *Anopheles* species complex [13]. Thus, the constraint of analyzing several selected genes, which requires sequence information, has made the molecular biology methods tedious, technically time-consuming and expensive approach to mosquito identification.

To overcome the drawbacks of molecular methods, the development of alternative tools has recently been explored. Based on the introduction of the matrix-assisted laser desorption/ionization time-of-flight mass spectrometry (MALDI-TOF MS) as an economic, rapid, and highly informative tool for bacterial identification and classification [14], this proteomic approach was explored as a taxonomic tool for insects. Since a pioneering study that evaluated MALDI-TOF MS for the discrimination of fruit fly species a decade ago [15], this technique has proven to be applicable for the identification of different arthropod groups, including *Drosophila* [16], *Culicoides* [17-20], *Ixodidae* [21,22], *Glossina* [23,24], *Phlebotominae* [25] and *Siphonaptera* [26]. MALDI-TOF MS was also successfully applied for the identification of adult mosquitoes allowing the discrimination of cryptic species such as the *An. gambiae* M and S molecular forms (Culicidae) [27-29]. More recently, the unambiguous identification of *Aedes* species based on mosquito egg protein profiling highlighted the robustness of MALDI-TOF MS for classification of mosquitos at even the pre-hatching developmental stage [30]. Nevertheless, until now, this technique has received scant attention for the identification of mosquitoes at their larval and pupal stages.

Therefore, the aim of the present study was to investigate the applicability of MALDI-TOF MS for the rapid identification of mosquito species at aquatic developmental stages. A simple protocol was optimized taking into account various parameters, including sample preparation, storage conditions, diet and body parts used. The goal was to establish a reference MS spectra database for mosquitoes from *Anopheles, Aedes* and *Culex* genuses at larval and pupal stages. The accuracy of this method in species identification according to developmental stages was then blindly evaluated.

## Methods

### Culicidae

Mosquitoes were reared in the URMITE laboratory (Marseille, France) or in the Institute Pasteur (Paris, France) using standard methods with temperature of 26 ± 1°C, a relative humidity of 80 ± 10% and a 12 h:12 h (light:dark) photoperiod in incubators (Panasonic cooled incubator) [31]. Larvae were reared until the pupal stage in trays containing 1 liter distilled water supplemented with fish food (TetraMinBaby, Tetra Gmbh, Herrenteich, Germany) or yeast tablets (Gayelord Hauser, Mequon, WI, USA). Pupae were daily collected and transferred to a mosquito cage (Bug Dorm 1, Bioquip products). Adults were fed with a 10% glucose solution. For eggs production, blood-meals were given through a Parafilm-membrane (hemotek membrane feeding systems, Discovery Workshops, UK) using fresh heparinized human blood or by providing anesthetized mice [32]. Seven mosquito colonies from 6 different species including *An. gambiae* (S form), *An. coluzzii* (M form), *Ae. albopictus (*two different colony origins), *Ae. aegypti*, *Cx. pipiens pipiens* and *Cx. pipiens molestus* were used to establish Culicidae juvenile database (Table 1). Specimens were collected from the L2 to L4 larval stages and pupal stage. Subsequently, the specimens were rinsed 60 sec. with 70% ethanol and 60 sec. with distilled water. The specimens were then directly treated for MALDI-TOF analyses or either frozen at −20°C or stored in 70% ethanol. Some specimens were dissected to compare protein mass profiles.

**Table 1:** **Culicidae used to establish the reference database of MALDI-TOF spectra and arthropods used in the blind test**

**Species**	**Geographical origin**	Source*	**No. of specimens used to create the database**	**Developmental stage** ^**a**^	**No. of specimens used for the blind test procedure**	**Log score-values** ^**b**^ **[low-High]**	**Stages identified**
*An. gambiae*(S form)	Montpellier, France (IRD)	UR	6	L2	11	[2.122 - 2.648]	L2, L3
			6	L3	15	[2.173 - 2.548]	L2, L3, L4
			6	L4	16 (2^c^)	[2.221 - 2.433]	L3, L4
			5	P1	12	[2.024 - 2.379]	P1
*An. coluzzi*	Dakar, Sengal	UR	6	L2	6	[2.196 - 2.466]	L2
			6	L3	5	[2.565 - 2.632]	L3
			6	L4	5	[2.325 - 2.499]	L3, L4
			6	P1	9	[2.177 - 2.588]	P1
*Ae. albopictus*	Montpellier, France (EID)	UR	6	L2	5	[2.023 - 2.435]	L2
			6	L3	12	[1.946 - 2.795]	L2, L3
			6	L4	14	[2.075 - 2.655]	L4, P1
			6	P1	5	[2.121 - 2.288]	P1
*Ae. albopictus*	Manaus, Brazil	PI	0	L3	5	[2.044 – 2.145]	L3
			0	L4	2	[1.987 - 1.995]	L4, P1
			0	P1	4	[1.940 - 2.239]	P1
*Ae. aegypti PAEA*	Papeete, Tahiti	PI	2	L2	1	[2.284]	L2
			2	L3	2	[2.241 - 2.468]	L2, L3
			2	L4	3	[1.958 - 2.383]	L4, P1
			2	P1	2	[2.394 - 2.441]	P1
*Cx. p. pipiens*		PI	2	L2			
			2	L3	1	[2.454]	L3
			2	L4	4	[2.143 - 2.419]	L3, L4
			2	P1	3	[2.086 - 2.516]	P1
*Cx. p. molestus*		PI	2	L2			
			2	L3	2	[2.049 - 2.11]	L2, L3
			2	L4	3	[2.504 - 2.03]	L3, L4
			2	P1	1	[2.528]	P1
**Total**			95		149		

*Location of laboratory-reared mosquitoes, UR, URMITE; PI, Pasteur Institute. ^a^Developmental stages are abbreviated as follows: L, larvae; P1, pupae collected at day 1. ^b^Range of identification log score values. ^C^Number of specimens misidentified at the species level.

### Larvae and pupae dissection

The heads and thoraces were separated from the abdomens of L3 stage *An. gambiae* and *Ae. albopictus* reared in the URMITE insectarium. Each body part of the specimen was then manually homogenized and treated with the standard procedure for MALDI-TOF analysis (for details see section “Preparation of samples for MALDI-TOF MS”).

### Feeding larvae

To test the effect of diet on MS profiles, hatched eggs from *An. gambiae* and *Ae. albopictus* reared in the URMITE insectarium were fed with 3 distinct diets, including fish food 1 (FF1) corresponding to standard diet (TetraMinBaby, Tetra Gmbh, Herrenteich, Germany), fish food 2 (FF2) containing a distinct composition (Tropical Mikrovit Basic, Tropical, Chorzów, Poland) or bread plus dried cat food (mix of beef and vegetables) (B + CF), during all their aquatic developmental stages. At the L3 stage, larvae were collected and treated for MALDI-TOF analyses.

### Preparation of samples for MALDI-TOF MS

Each whole mosquito specimen was homogenized in 20, 30, 40 and 50 μL of 70% formic acid for stages L2, L3, L4 and pupae, respectively. Homogenizations were performed manually using pestles (Fischer Scientific, Strasbourg, France) or with a FastPrep-24 Instrument (MP BIOMEDICALS, Santa Ana, California, USA) using glass beads (Sigma, Lyon. France). Suspensions were mixed with 50% acetonitrile (v/v) (Fluka, Buchs, Switzerland) and centrifuged at 10,000 rpm for 20 sec. One microliter of the supernatant of each sample was deposited on a steel target plate (Bruker Daltonics, Wissembourg, France) into four spots as previously described [28]. Then, 1 μL of CHCA matrix composed of saturatedα-cyano-4-hydroxycynnamic acid (Sigma, Lyon. France), 50% acetonitrile(v/v), 2.5% trifluoroacetic acid (v/v) (Aldrich, Dorset, UK) and HPLC-grade water was directly overlaid on each spot sample on the target plate, dried for several minutes at room temperature and introduced into the MALDI-TOF MS instrument for analysis. To control loading on mass spectra steel, matrix quality and MALDI-TOF apparatus performance, matrix solution was loaded in duplicate onto each MALDI-TOF plate with or without a bacterial test standard (Bruker protein Calibration Standard I).

### MALDI-TOF MS parameters

Protein mass profiles were obtained using a Microflex LT MALDI-TOF mass spectrometer (Bruker Daltonics, Germany), with detection in the linear positive-ion mode at a laser frequency of 50 Hz within a mass range of 2–20 kDa. The acceleration voltage was 20 kV, and the extraction delay time was 200 ns. Each spectrum corresponds to ions obtained from 240 laser shots performed in six regions of the same spot and automatically acquired using the AutoXecute of the Flex Control v.2.4 software (Bruker Daltonics). The spectrum profiles obtained were visualized with Flex analysis v.3.3 software and exported to ClinProTools software v.2.2 and MALDI-Biotyper v.3.0. (Bruker Daltonics, Germany) for data processing (smoothing, baseline subtraction, and peak picking) and evaluation with cluster analysis.

### Spectra analysis and reference database creation

Species spectra reproducibility at each aquatic developmental stage was evaluated by comparing the average spectra of each specimen within a species using the ClinProTools 2.2 software (Bruker Daltonics). To create a database for each aquatic developmental stage species, reference spectra (MSP, Main Spectrum Profile) were created by combining the results of the spectra from at least 2 to 6 specimens per developmental stage per species by the automated function of the MALDI-Biotyper software v3.0. (Bruker Daltonics). MSP were created on the basis of an unbiased algorithm using information on the peak position, intensity and frequency.

### MALDI-TOF MS biomarker mass set

To determine the species differential peaks from the samples of 2 *Aedes* spp, 2 *Culex* spp and 2 *Anopheles* spp tested (Table 1), a total of 128spectra from specimens at the L3 stages and day one of the pupal (P1) aquatic developmental stage of each species included in the database were loaded into ClinProTools2.2 software. The software was used to generate a peak list for each species in the 2 to 20 kDa mass range and to identify discriminating peaks among the analyzed species. The parameter sets in ClinProTools 2.2 software for spectra preparation were as follows: a resolution of 300; a noise threshold of 2.00; a maximal peak shift of 800 ppm and a match to calibrant peaks of 10%. For the peak calculation, peak peaking was performed on single spectra with a signal-to-noise threshold of 2.00 and an aggregation of 800 ppm. The spectra were then analyzed with the genetic algorithm (GA) model, which displayed a list of discriminating peaks. A manual inspection and validation of the selected peaks by the operator gave a “recognition capability” (RC) value together with the highest “cross-validation” (CV) value. The presence or absence of all discriminating peak masses generated by the GA model was controlled by the comparison of the average spectra from each species at the L3 and P1 stages.

### Blind tests for study validation

The reference spectra of each species at different developmental stages were evaluated using a blind test performed with new specimens at different aquatic developmental stages from laboratory-reared mosquito colonies. The level of significance identification was determined using the log score values (LSVs) given by the MALDI-Biotyper software v.3.3. corresponding to a matched degree of signal intensities of mass spectra of the query and the reference spectra. LSVs ranged from 0 to 3. A LSV for species identification and developmental stage was obtained for each spectrum of the samples tested blindly.

### Cluster analysis

Cluster analysis (MSP dendrogram) was performed based on the comparison of the main spectra given by MALDI-Biotyper software and clustered them according to the protein mass profile (*i.e.,* their mass signals and intensities). Several clustering analyses were performed to determine how organisms are related to one another.

### Ethical approval for animal use

The Institut Pasteur animal facility has received accreditation from the French Ministry of Agriculture to perform experiments on live animals in compliance of the French and European regulations on care and protection of laboratory animals. This study was approved by the Institutional Animal Care and Use Committee (IACUC) at the Institut Pasteur. No specific permits were required for the described field studies in locations that are not protected in any way and did not involve endangered or protected species.

## Results and discussion

### Evaluation of sample preparation parameters for MALDI-TOF profiling

#### Whole vs. body parts and diet

**Figure 1:**
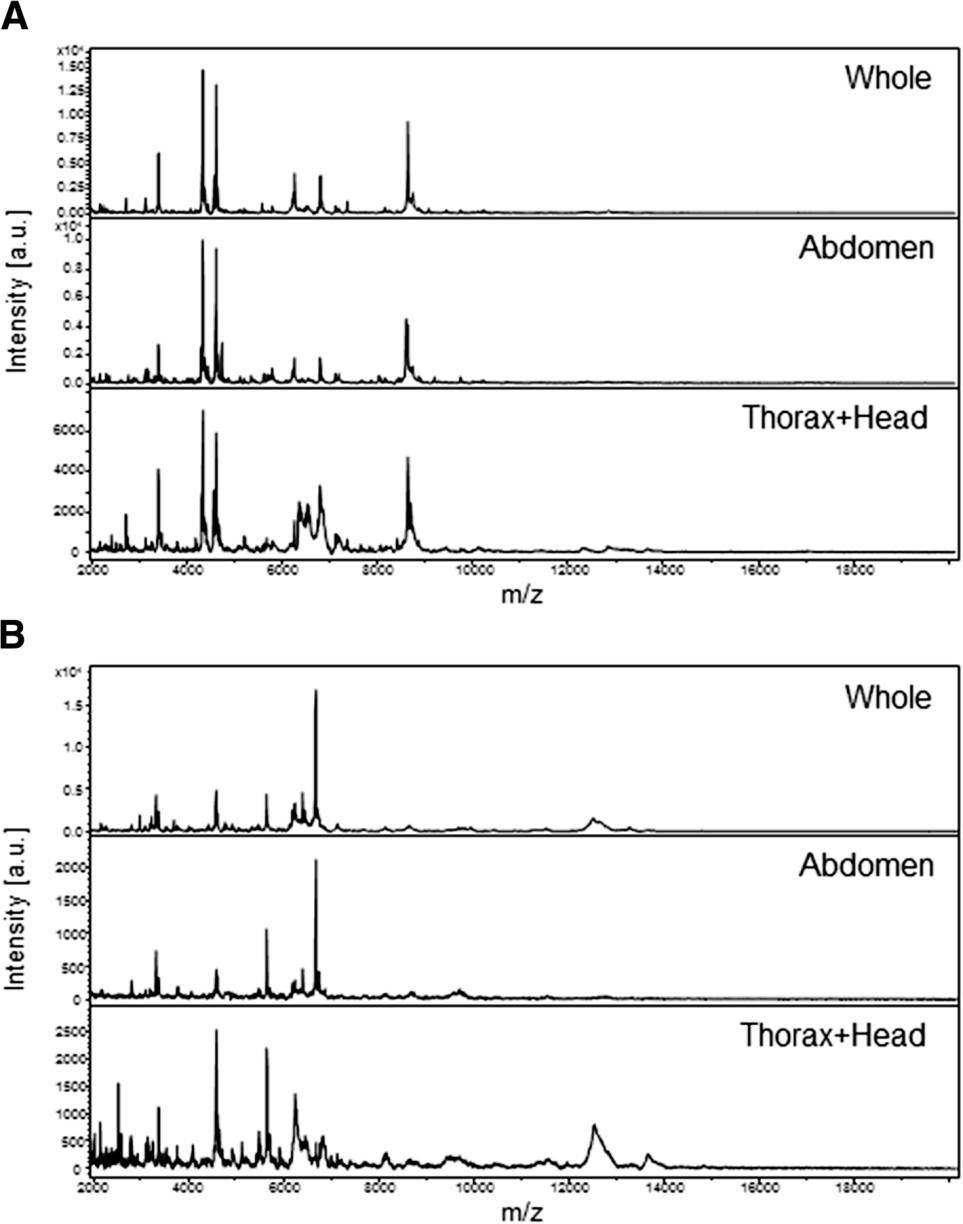
**Comparison of MALDI-TOF MS spectra of whole specimens or body parts of mosquitoes at the L3 stage ranging from 2 to 20 kDa.** Representative spectra from biological replicates performed in quadruplicate of whole (top-spectra), abdomen (middle-spectra) and thorax + head (bottom- spectra) body parts from *An. gambiae* specimens **(A)** and *Ae. albopictus* **(B)** are shown. The mosquito body parts are indicated in the right corner of each protein profile spectrum. a.u., arbitrary units; m/z, mass-to-charge ratio.

To determine whether abdomen compartment could affect negatively protein profiling analysis and to estimate consequences of gut contents according to distinct dietary feeding, a comparison of MALDI-TOF protein profiles obtained for whole specimens and body parts (*i.e.,* abdomen or thorax plus head) from *An. gambiae* and *Ae. albopictus* at the L3 larval stage was performed. At least four biological replicates were tested for each condition using these two mosquito species reared at the URMITE insectarium. For *An. gambiae*, MS profiles generated from whole specimens and abdomens were more reproducible amongst themselves than compared to thorax plus head (Figure 1A). Similar results were obtained for MS profiles generated from whole specimens and body parts of *Ae. albopictus* specimens (Figure 1B). Interestingly, abdomen protein profiles were highly distinct between *Ae. albopictus* and *An. gambiae* specimens despite similar diets (*i.e.,* fish food (TetraMin)). Additionally, the protein profiles for each of these two mosquito species were reproducible for either whole insects or body parts at the L3 larval stage collected at a one month interval (data not shown). The similarity of the MS profiles between whole specimens and abdomens suggested that the prominent peaks may correspond to abdomen protein origins. MALDI-TOF MS detects mainly the most abundant proteins and peptides of low molecular weights (*i.e.,* ranging from 2 to 20 kDa) [14]. Although the whole protein repertoire is specific for each body part of an organism, it has been demonstrated that some body parts from the same insect generate similar MALDI-TOF MS profiles [17].

**Figure 2:**
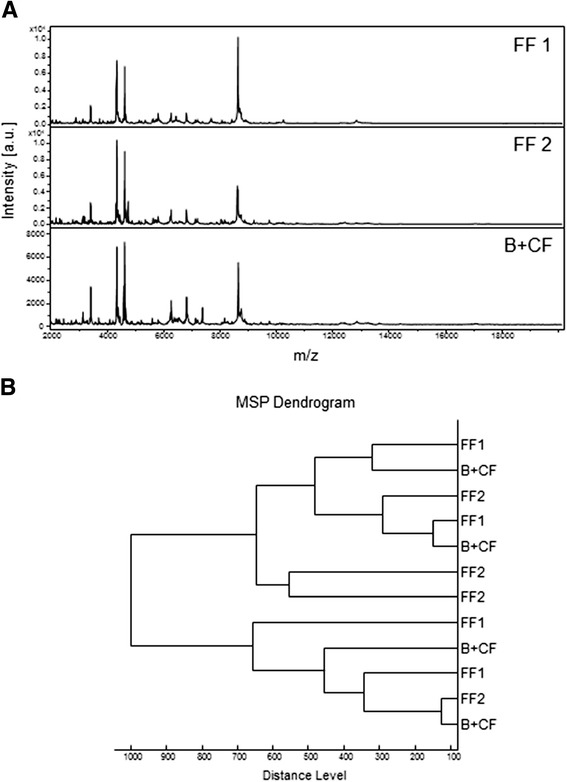
**Consequences of diet on MALDI-TOF MS profiles from whole** ***An. gambiae*** **specimens at the L3 stage. (A)** Representative spectra from biological replicates performed in quadruplicate of *An. gambiae* specimens fed with fish food 1 (top-spectra), fish food 2 (middle-spectra), or bread plus dried cat food (bottom-spectra) during all aquatic developmental stages. The diets provided are indicated in the right corner of each protein profile spectrum. a.u., arbitrary units; m/z, mass-to-charge ratio. **(B)** Dendrogram constructed from 2 representative spectra of *An. gambiae* specimens fed with fish food 1, fish food 2 or bread plus dried cat food. The dendrogram was calculated by Biotyper 3.0 software and the distance units correspond to the relative similarity of MS spectra. FF1, fish food 1 (TetraMinBaby); FF2, fish food 2 (Tropical Mikrovit Basic); B + CF, bread plus dried cat food.

It has been repeatedly reported that gut contents may impair protein profiles, notably for adult hematophagous arthropods corresponding to blood-feeding behavior [19,21]. The abdomen is then generally dissected and excluded prior MALDI-TOF analysis. To determine the consequences of food onto MALDI-TOF MS profiles from whole L3 specimens, *An. gambiae* and *Ae. albopictus* were fed with three distinct diets (*i.e.,* fish food1 (FF1, TetraMinBaby), fish food 2 (FF2, Tropical Mikrovit Basic) or bread plus dried cat food (B + CF)) during all aquatic developmental stages. The MS profiles from whole L3 specimens were very similar regardless of the diet provided and according to the mosquito species (Figure 2A). Moreover, 4 representative MS profiles of *An. gambiae* specimens at the L3 larval stage per dietary condition were used to perform clustering analysis. Inconsistent clustering was obtained according to food delivered as observed in the dendrogram (Figure 2B). Comparable results were obtained for *Ae. albopictus* specimens at the L3 larval stage fed with these three distinct diets (data not shown). These results revealed that MS protein profiles were weakly affected by diet.

Taken together, the reproducibility of the whole and abdomen protein profiles at the L3 larval stage and their distinction between *An. gambiae* and *Ae. albopictus* specimens regardless of the method of generation and diet underlined that gut protein contents appear to have a minor effect on MS protein patterns. As the final objective was to develop a novel fast, simple and accurate method that allows low sample handling to identify Culicidae at the aquatic developmental stages, the use of whole specimens was chosen for the next parameters tested and to create a spectra library database for the further evaluation of Culicidae identification.

#### Developmental stages from L2 to L4 and pupal

As mosquitoes are holometabolous insects, during their aquatic developmental stages a metamorphosis occurs. Insect metamorphosis has already been described to induce protein repertoire changes [33]. Although MALDI-TOF MS has been shown to be applicable for the identification and discrimination of several adult insect species, scant attention has been paid to the assessment of this tool for the identification of arthropod species at aquatic life cycle stages. Only some species of ticks and biting midges have been previously analyzed with MALDI-TOF profiling that took into account developmental stages [20,21]. Distinct MS profiles were observed for ticks at each developmental stage (*i.e.,* eggs, larvae, nymphs or adults) [21]. For Culicoides biting midges, modifications of MS profiles occurred during their metamorphosis that distinguish the larval, pupal and adult specimens [20]. More recently, fleas also showed an evolution of protein profiles according to their developmental stages [26].

**Figure 3:**
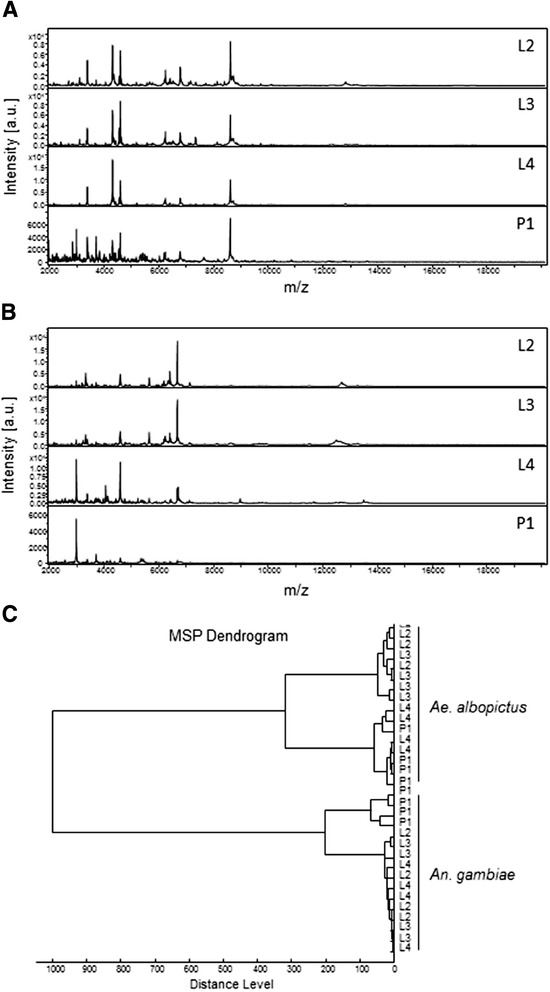
**MALDI-TOF MS spectra of whole mosquitoes at different aquatic stages ranging from 2 to 20 kDa.** A representative spectra from biological replicates performed in quadruplicate of each aquatic developmental stage of *An. gambiae* **(A)** and *Ae.albopictus* **(B)** specimens is shown. The mosquito life stages are indicated in the right corner of each protein profile spectrum. a.u., arbitrary units; m/z, mass-to-charge ratio; L2 to L4, larval stages 1 to 4; P1, pupae at day 1. **(C)** Dendrogram of MALDI-TOF MS spectra of different life stages of *An. gambiae* and *Ae. albopictus*. Each life stage is represented by 2 distinct specimens. Distance unit correspond to the relative similarity calculated from the distance matrix.

Therefore, the MS protein profiles from entire specimens according to their developmental stages were compared for *An. gambiae* and *Ae. albopictus* specimens. For *An. gambiae*, MS protein profiles were weakly modified from the L2 to L4 developmental stages (Figure 3A). Conversely, at the pupal stage, the patterns changed dramatically. Concerning *Ae. albopictus* specimens, from the L2 to L3 developmental stages, MS profiles were stable, whereas their L4 and pupal stages were modified (Figure 3B). To visualize the distances between the different aquatic stages and species, MS protein profiles of two specimens per developmental stage from these two mosquito species were used to generate a dendrogram (Figure 3C). Clustering analyses were therefore performed with specimens from the L2 to L4 larval stages and the day-one pupal stage (P1). Clustering analysis revealed that all stages of the same species gathered on distinct branches and no overlapping occurred between the two mosquito species tested. For *An. gambiae*, a clear-cut separation was detected between the pupal and larval stages. However, intertwining occurred between the different larval stages, suggesting a low specificity of MS profiles according to larval stages. Concerning *Ae. albopictus* specimens, early larval stages (*i.e.,* L2 and L3) clustered together and were separated from the L4 and pupal stages which shared the same main branch. Then, the protein pattern changes that occurred at the late larval and pupal stages reflect the biologic metamorphosis of the mosquitoes. Interestingly, these modifications arise at an earlier aquatic life cycle in *Aedes* than in *Anopheles* mosquitoes. This cluster analysis on the basis of MALDI-TOF MS indicated that the primary determinant for the MS profiles was the species, followed by the clustering of early *vs*. late aquatic developmental stages.

#### Sample preparation and storage methods (fresh, 70% ethanol, frozen, in water)

To standardize the homogenization of immature stages, a comparison of the MS profiles obtained from a disruption of tissues by hand with a pestle or by an automated beater system using glass beads (*i.e.,* FastPrep-24 Instrument) was tested. Both methods of sample preparation yielded MS profiles of equal quality (Additional file 1: Figure S1A and S1B). Thus, for the case in which a large number of specimens must be identified by MALDI-TOF MS, the automation of sample homogenization, which is less laborious, is conceivable.

As whole specimen homogenization in formic acid degrades DNA, the effect of manual disruption of *An. gambiae* in sterile distilled water on MS profiles was tested (Additional file 1: Figure S1C). This homogenization method is compatible with DNA extraction for the eventual future validation of identification by molecular biology. With the exception of the decreased intensity of one peak (*i.e.,* 8641.4 kDa), MS patterns were reproducible. Water can then be used for both MALDI-TOF MS and DNA isolation analysis as previously described [17,19].

The collection sites and laboratory locations for the monitoring of field mosquitoes at aquatic stages using MS analysis could be separated by variable distances. Thus, different modes of storage of immature stages were investigated. L3 larvae of *An. gambiae* mosquitoes were stored for different time periods (*i.e.,* 7, 14, 21, 28 and at least 60 days) frozen or in 70% ethanol at room temperature. Storage up to two months at −20°C had no deleterious effect on the MS profiles (Additional file 1: Figure S1D). Conversely for mosquito larvae stored in ethanol, the intensity of some peaks was modified, and background noise was noticeable and appeared linked to the length of time in storage (Figure 1E-G). Storage in ethanol has already been reported to be deleterious for arthropod MALDI-TOF MS analysis [21,26]. Thus, freezing is the recommend mode of storage when mosquito aquatic stages cannot be analyzed immediately.

Taken together, the recommended procedure for MALDI-TOF MS analyses of Culicidae aquatic stages is to homogenize the entire specimen manually or automatically, either in water or formic acid. Moreover, storage by freezing appears better than the 70% ethanol preservation mode.

### MALDI-TOF MS analysis and reference spectra database assembly

**Figure 4:**
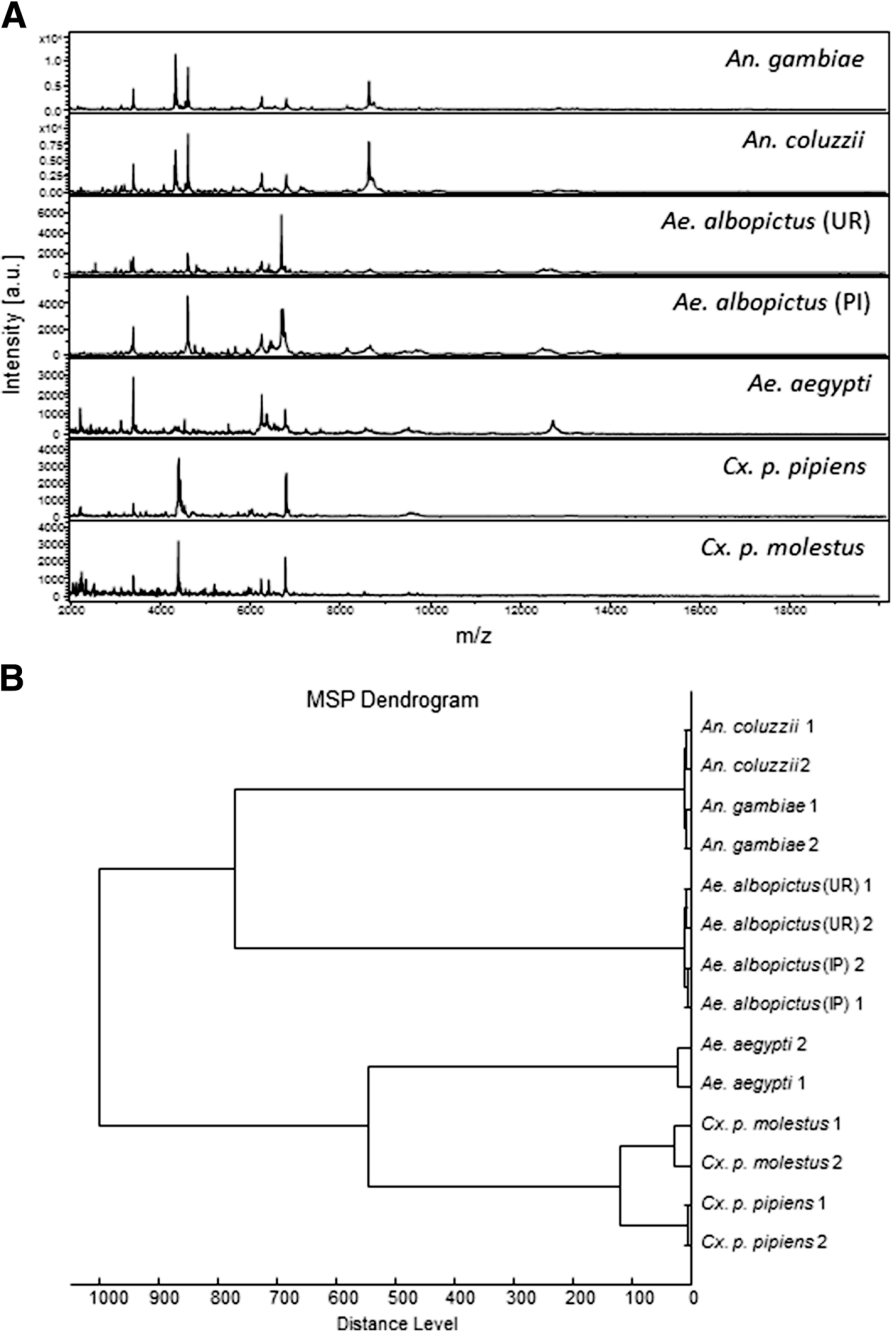
**MALDI-TOF MS spectra of whole mosquito specimens at the L3 stage from six different species ranging from 2 to 20 kDa. (A)** A representative spectra from biological replicates performed in quadruplicate of each mosquito species is shown. The mosquito species is indicated in the right corner of each protein profile spectrum. a.u., arbitrary units; m/z, mass-to-charge ratio; L3, larval stage 3. **(B)** Dendrogram of MALDI-TOF MS spectra from the six mosquito species. Each species is represented by 2 distinct specimens. Distance unit correspond to the relative similarity calculated from the distance matrix.

The MALDI-TOF MS approach to identify mosquito species at aquatic stages was assessed. A total of 95 mosquito specimens from 6 species at 4 distinct aquatic developmental stages (*i.e.,* from L2 to L4 and pupae at day 1), including at least 2 species from the 3 main Culicidae genera (*i.e., Anopheles, Aedes* and *Culex*), were subjected to MALDI-TOF MS analysis. As observed for *An. gambiae* and *Ae. albopictus*, the MS spectra of each species according to developmental stage were reproducible after spectral analysis and alignment. The alignment of representative spectra from the different species tested at the L3 larval developmental stage is presented in Figure 4A. On average, 96 peaks were detected per spectrum at L3, ranging between 59 and 116 peaks using ClinProTools. At the P1 stage, 70 peaks ranging from 52 to 83 were retrieved.

The cluster analysis using two main spectra of each species at the L3 larval stage including the two colonies of *Ae. albopictus* is shown in Figure 4B. All specimens were correctly classified at the species level and no inter-species overlapping was detected. Despite the similarity of some profiles, notably for cryptic species such as *Cx. p. pipiens* and *Cx. p. molestus* or *An. gambiae* and *An. coluzzii*, each species clustered on discrete branches. Moreover, the two colonies of *Ae. albopictus* from different geographical origins clustered together but were found in distinct branches. Surprisingly, the mosquitoes from the *Aedes* genus (*i.e., Ae. albopictus* and *Ae. aegypti*) did not cluster in the same region of the dendogram. These results indicated that, despite the correct classification of the specimens at the species level, MALDI-TOF MS does not seem to be a relevant tool for phylogenetic analysis as previously described [21,29].

To identify discriminatory peaks among the 6 mosquito species analyzed in the present study, 16 spectra per species at the L3 and P1 stages were analyzed using the Genetic Algorithm (GA) tool from ClinProTools software. After verification of the peak report in the average spectrum of the 6 mosquito species tested, 35 and 23 discriminatory masses were determined at L3 and P1, respectively (Tables 2 and 3). The combination of the presence/absence of these discriminant peaks displayed RC and CV values of 100% at the L3 stage. At the P1stage, RC and CV values of 100% and 99.24%, respectively, were obtained. Subsequently, two to six specimens at the 4 developmental stages (*i.e.,* L2 to L4 and P1) and per species were used to create a MS reference database (Table 1).

**Table 2:** **Discriminating mass peaks between the six Culicidae species included in the reference MS database of MALDI-TOF at the L3 aquatic stage**

**Number**	**Mass m/z [Da]**	**Start mass m/z [Da]**	**End mass m/z [Da]**	***An. gambiae***	***An. coluzzii***	***Ae. aegypti***	***Ae. albopictus***	***Cx. p. pipiens***	***Cx. p. molestus***
**1**	2213.63	2204.71	2220.92	-	-	-	-	+	+
**2**	2227.55	2226.53	2235.88	+	+	-	-	-	-
**3**	2709.15	2702.45	2719.88	+	+	-	-	-	-
**4**	3002.61	2991.93	3005.19	-	+	-	+	-	-
**5**	3198.93	3187.73	3204.2	-	+	-	-	-	-
**6**	3344.75	3337.51	3354.32	-	-	-	+	-	-
**7**	3460.33	3451.2	3472.71	+	+	-	-	-	-
**8**	3724.44	3711.33	3735.47	+	+	-	+	-	-
**9**	4074.85	4063.87	4087.13	-	+	-	-	-	-
**10**	4331.99	4321.54	4346.68	+	+	-	-	-	-
**11**	4401.47	4398.34	4417.65	-	-	-	-	+	+
**12**	4431.45	4418.55	4435.31	-	-	-	-	+	+
**13**	4461.79	4449.63	4471.79	-	-	-	-	+	+
**14**	4532.78	4518.9	4547.36	-	-	-	-	+	-
**15**	4607.44	4588.57	4609.64	+	+	-	+	-	-
**16**	4668.7	4659.29	4680.67	+	-	-	-	-	-
**17**	4942.9	4927.38	4957.08	-	-	-	+	+	+
**18**	5193.44	5180.07	5200.56	+	+	-	-	-	-
**19**	5211.24	5200.56	5228.38	+	+	-	-	-	-
**20**	5574.68	5560.73	5593.11	+	-	-	-	-	-
**21**	5615.54	5597.96	5632.36	-	+	+	-	-	-
**22**	5662.87	5652.69	5667.51	-	-	-	+	-	-
**23**	5734.88	5718.69	5751.92	-	-	-	-	+	-
**24**	5981.4	5961.06	5993.71	-	-	+	-	+	-
**25**	6031.11	6022.16	6051.79	-	-	-	-	+	-
**26**	6423.55	6416.53	6426.92	-	-	-	+	-	-
**27**	6458.42	6445.18	6478.9	-	-	-	+	-	-
**28**	6691.94	6669.95	6713.95	-	-	-	+	-	-
**29**	6791.36	6774	6794.05	-	-	+	+	-	-
**30**	6797.41	6794.05	6817.84	+	+	-	-	+	+
**31**	7115.51	7095.02	7129.98	+	+	-	-	-	-
**32**	7558.82	7541.81	7580.28	-	-	+	-	-	-
**33**	8633.2	8600	8648.73	+	+	-	-	-	-
**34**	8690.6	8676.18	8721.32	+	-	-	-	-	-
**35**	9070.26	9045	9102.62	-	+	-	-	-	+
**Total**				**14**	**16**	**4**	**10**	**10**	**7**

**Table 3:** **Discriminating mass peaks between the six Culicidae species included in the reference MS database of MALDI-TOF at the P1 aquatic stage**

**Number**	**Mass m/z [Da]**	**Start mass m/z [Da]**	**End mass m/z [Da]**	***An. gambiae***	***An. coluzzii***	***Ae. aegypti***	***Ae. albopictus***	***Cx. p. pipiens***	***Cx. p. molestus***
**1**	2999.89	2990.67	3002.65	-	-	+	+	+	+
**2**	3026.17	3025.58	3036.29	-	-	-	-	+	+
**3**	3437.1	3433.23	3448.71	+	+	-	-	-	-
**4**	3514.66	3501.34	3526.44	-	-	-	+	-	-
**5**	3593.03	3586.61	3601.84	-	+	-	-	-	-
**6**	4020.58	4006.76	4031.99	+	+	-	-	-	-
**7**	4209.88	4197.71	4215.43	-	-	-	-	-	+
**8**	4225.44	4215.43	4231.37	-	+	-	-	-	-
**9**	4240.36	4231.37	4244.23	-	-	-	+	+	-
**10**	4280.13	4270.75	4297.96	-	-	-	-	-	+
**11**	4330.76	4321.31	4350.08	+	+	-	-	-	-
**12**	4398.69	4393.23	4416.32	-	-	-	-	+	+
**13**	4558.05	4545.6	4566.28	+	+	-	-	+	-
**14**	4607.52	4603.55	4620.78	+	+	-	+	-	-
**15**	4645.98	4639.27	4660.39	-	-	-	-	-	+
**16**	5157.09	5148.73	5173.1	-	-	+	-	-	-
**17**	5435.44	5413.08	5437.56	-	-	-	-	+	+
**18**	5663.42	5651.19	5685.79	-	-	-	+	-	-
**19**	5915.95	5905.68	5930.73	-	+	+	+	+	+
**20**	6347.7	6336.1	6366.85	+	-	-	-	-	-
**21**	6464.08	6443.81	6467.51	-	-	-	+	-	-
**22**	8633.57	8597.27	8664.94	+	+	-	-	-	-
**23**	10873.74	10844.92	10893.96	+	+	-	-	-	-
**Total**				**8**	**10**	**3**	**7**	**7**	**8**

### Validation step

The reproducibility and accuracy of the reference database were tested in a validation study. Then, 149 Culicidae specimens at the four aquatic developmental stages (from L2 to L4 and P1) from the sic mosquito species were subjected to MALDI-TOF MS analysis. The resulting spectra were queried against the MS reference database yielding more than 98% (n = 147/149) correct identification at the species level regardless of the developmental stage with identification LSVs between 1.940 and 2.795 (Table 1).

Lower LSVs were obtained for *Ae. albopictus* specimens reared at the Pasteur Institute than those collected at Manaus in Brazil. In the reference spectra database, only *Ae. albopictus* specimens from aquatic stages reared at the URMITE insectarium were included. Thus, despite the correct identification, these lower LSVs could be attributed to intra-species MS profile variations from specimens coming from distinct geographical origins. The detection of spectral differences for arthropods from the same species stemming from distinct locations has already been reported in tsetse flies [24] and in phlebotomine sand flies [25]. These MS profile variations could result from confounding effects between environmental and biologic factors. However, the correct identification of *Ae. albopictus* with high LSVs suggests that MALDI-TOF MS tools may be useful for the identification of other colonies from this same species at aquatic stages. Reliable identification is particularly important as *Ae. albopictus* have colonized every continent except Antarctica [34]. The widespread distribution of this mosquito species, a vector of arboviruses of public health significance, makes the monitoring and control of *Ae. albopictus* the best disease prevention method [5,35]. Therefore, the rapid identification of aedine species at aquatic stages may be helpful for the monitoring and control of this invasive mosquito species and the precise targeting of breeding sites. These results suggest that the implementation of the MS reference database with spectra from specimens of species already included in the database but collected in distinct areas (*i.e.,* specimens from the area of observation) seems a valid initiative to improve identification of local mosquitoes.

Mosquitoes from the *Cx. pipiens* complex include 6 members, such as *Cx. pipiens pipiens* and *Cx. p. molestus*, which exhibit different physiological and behavioral traits that greatly influence their vectorial capacity [36,37]. Moreover, *Cx. p. pipiens* and *Cx. p. molestus* subspecies are morphologically similar and their identification, in temperate latitudes, has been associated particularly in differences of larval habitats [37]. Therefore, the development of a new tool for distinguishing the two mosquitoes from the *Cx. pipiens* complex at aquatic stages would be indispensable. Here, there was no ambiguity in their identification by applying the MALDI-TOF MS strategy.

Finally, among the entire specimens tested, only two were incorrectly identified at the species level (Table 1). Two *An. gambiae* specimens at the L4 stage were identified as *An. coluzzii* at the L3 stage as the top-ranking hit. This misidentification may be attributed to the low diversity of the MS spectra between these two *Anopheles* species belonging to the same complex. The accurate paired comparison of MSP from *An. gambiae* and *An. coluzzii*, indicated that 8 and 4peaks could distinguish them at the L3 and P1 aquatic developmental stages, respectively. Although 97.3% (73/75) of the specimens from this *Anopheles* complex tested blindly were unambiguously distinguished, additional experiments notably using *Anopheles* specimens collected in the field are needed to confirm the utility of MALDI-TOF MS for the reliable classification of these species at their aquatic stages. Moreover, it is possible that the combination of the low diversity of MS spectra between these two species and the intra-species profile variations due to geographical origins may alter the distinction of these two *Anopheles* molecular forms at aquatic stages. Nevertheless, the discrimination of *An. coluzzii* from *An. gambiae* has been previously demonstrated to be possible at the adult stages by MALDI-TOF MS [27,29]. Therefore, confirmation is possible with larval specimens collected at the same breeding site, reared in the laboratory until adult stage and submitted to MALDI-TOF MS. Alternatively, it would also be possible to utilize previously discussed molecular strategies in parallel with MALDI-TOF MS analysis in cases of uncertainty identification.

Interestingly, more than 80% of the specimens were correctly identified at their respective developmental stage level. The imprecision in the aquatic stage determination is ascribed to the sharing of numerous MS peaks in the course of their early (*e.g.,* L2 or L3) or late (*e.g.,* P1) life cycle. Specific MS signatures could be then attributed to early and late aquatic developmental stages. Thus, the addition of some reference spectra from early (*i.e.,* L2 or L3) and late (*i.e.,* P1) stages of a new species would be sufficient for their future identification.

To control for the accuracy of juvenile mosquito identification by MALDI-TOF MS, these 149 specimens were also tested blindly against our in-house arthropod database composed of several species of mosquitoes (n = 30), fleas (n = 5) and ticks (n = 6) at adult stages [22,26,28,29]. The highest LSV obtained was less than 1.5, which corresponds to a misidentification and underlines the absence of cross-recognition.

## Conclusions

Beyond the demonstration that MALDI-TOF MS could successfully identify distinct mosquito species from three genera at the juvenile stages, the present work emphasized that this proteomic tool may also reveal distinct cryptic species, molecular forms from a complex species and distinct mosquito species according to geographical origin. Despite the lack of exact determination of developmental stage due to the conservation of MS profiles during part of the mosquito aquatic life cycle, it is possible to define whether a specimen is in the early or late part of its developmental aquatic stage. The quick identification of juvenile mosquitoes by MALDI-TOF MS could be an alternative tool to monitor Culicidae vectors. It now becomes conceivable to perform accurate “live” monitoring of mosquito breeding sites and implement anti-vectorial measures according to the mosquito fauna detected prior to their emergence.

## Additional file

Additional file 1: Figure S1. Effect of sample preparation methods and storage conditions on the spectra quality. Comparison of the MALDI-TOF MS spectra of whole *An. gambiae* at the L3 stage homogenized manually with a pestle (A), automatically with a FastPrep apparatus (B), manually in deionized water followed by acidic extraction (C); stored at −20°C for 60 days and homogenized manually with a pestle (D); stored in 70% ethanol for 7 (E), 14 (F) and 60 (G) days. a.u., arbitrary units; m/z, mass-to-charge ratio.
