# Supplementary figures and images for: Accurate identification of Culicidae at aquatic developmental stages by MALDI-TOF MS profiling
Source: Parasit Vectors. 2014 Dec 2;7:544. doi: 10.1186/s13071-014-0544-0 (PMC4273427; doi:10.1186/s13071-014-0544-0)

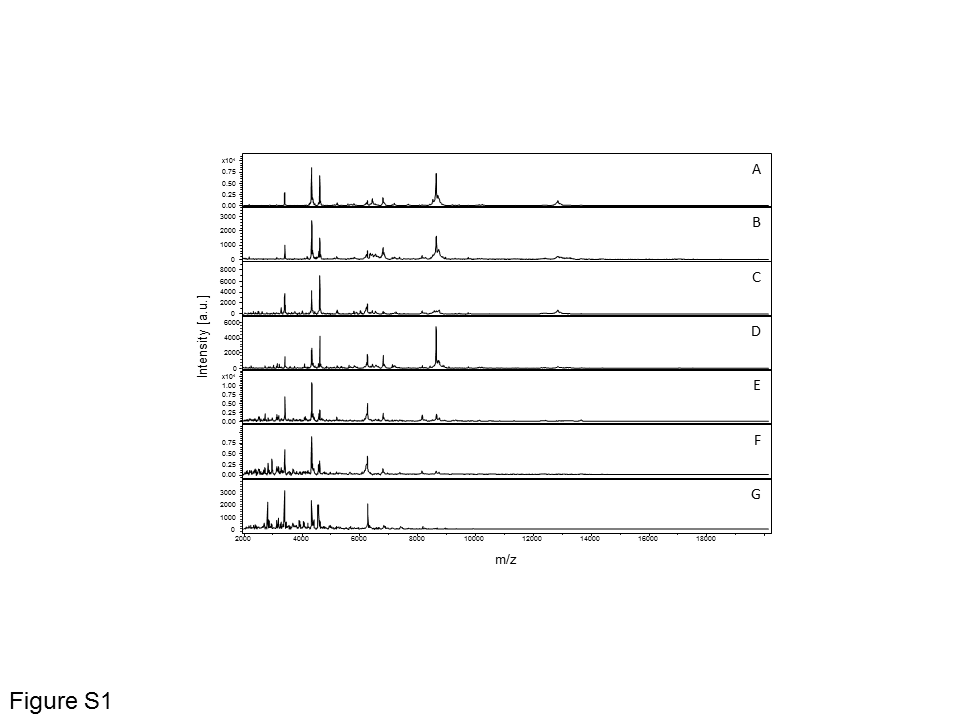

Supplement: Additional file 1: Figure S1. — Effect of sample preparation methods and storage conditions on the spectra quality. Comparison of the MALDI-TOF MS spectra of whole An. gambiae at the L3 stage homogenized manually with a pestle (A), automatically with a FastPrep apparatus (B), manually in deionized water followed by acidic extraction (C); stored at −20°C for 60 days and homogenized manually with a pestle (D); stored in 70% ethanol for 7 (E), 14 (F) and 60 (G) days. a.u., arbitrary units; m/z, mass-to-charge ratio. [file 13071_2014_544_MOESM1_ESM.tiff]
